# Circulating chemerin levels and gestational diabetes mellitus: a systematic review and meta-analysis

**DOI:** 10.1186/s12944-018-0826-1

**Published:** 2018-07-24

**Authors:** Zhongwei Zhou, Hongmei Chen, Huixiang Ju, Mingzhong Sun

**Affiliations:** Department of Clinical Laboratory, Affiliated Yancheng Hospital, School of Medicine, Southeast University, Jiangsu 224001 Yancheng, People’s Republic of China

**Keywords:** Chemerin, Gestational diabetes mellitus, Insulin resistance, Systemic inflammation, Meta-analysis

## Abstract

**Background:**

Chemerin is a novel adipokine which is associated with metabolic syndrome and type 2 diabetes mellitus. However, recent investigations regarding circulating chemerin levels in gestational diabetes mellitus (GDM) are conflicting. This meta-analysis is to evaluate and determine their associations.

**Methods:**

A systematic literature search was performed in PubMed, EMBASE and Web of Science up to 13 December 2017. Pooled standardized mean differences (SMD) and 95% confidence interval (CI) were calculated using a random-effect model.

**Results:**

Eleven studies comprising 742 GDM patients and 840 normal pregnant women were included. Circulating chemerin levels were increased in GDM patients compared with healthy pregnant women (SMD: 1.16; 95% CI: 0.29, 2.04; *P* = 0.009). Subgroup analyses revealed such difference was especially available in the groups of the second trimester (SMD: 1.47; 95% CI: 0.28, 2.67) and mean age < 30 years (SMD: 2.30; 95% CI: 0.69, 3.91) of GDM patients. There was significant heterogeneity among studies (*I*^2^ = 98.0%, *P* < 0.001); however, heterogeneity disappeared or markedly decreased in the subgroups of European populations (*I*^2^ = 0.0%, *P* = 0.531), age ≥ 30 years (*I*^2^ = 28.2%, *P* = 0.223) and WHO diagnostic criteria (*I*^2^ = 0.0%, *P* = 0.490) when stratifying by study location, trimester of chemerin measurement and the diagnostic criteria of GDM.

**Conclusions:**

The elevated levels of circulating chemerin were associated with GDM, which suggests it might play an important role in the pathogenetic mechanism of GDM.

## Background

Gestational diabetes mellitus (GDM) is defined as varying degrees of glucose intolerance first detected during pregnancy, which affects 4–18% pregnant women according to different diagnostic criteria and ethnic origin [1, 2]. The pathophysiologic mechanism of GDM is similar to type 2 diabetes mellitus (T2DM), including insulin resistance, oxidative stress and systemic inflammation [3]. It is also suggested that pregnant women who develop GDM may have pre-existing β-cell defects unable to adapt to the increasing demands of insulin during pregnancy [4, 5], and now it is widely believed that systemic inflammation associated with β-cell dysfunction and the subsequent insulin resistance in diabetic patients [6, 7]. So, whatever view one takes, it is undeniable that insulin resistance and chronic low-grade inflammation play vital roles in the progression of GDM.

Chemerin is a novel cytokine mainly secreted from white adipose tissues, which was initially considered as a chemotactic factor generated in inflammatory conditions, but more recently, it was reported more as an adipokine regulating metabolism of adipose and balance of energy [8, 9]. Serum levels of chemerin were shown to be markedly elevated in patients with biopsy-proven non-alcoholic fatty liver disease (NAFLD) compared with healthy controls [10], and elevated hepatic chemerin mRNA expression also was confirmed to be independently associated with liver fibrosis, steatosis, inflammation, and hepatocyte ballooning in human NAFLD [11]. A recent population-based study revealed that increased chemerin levels were associated with inflammation and metabolic syndrome even after adjustment for waist circumference [12]. More importantly, chemerin has been indicated to be an independent predictor of type 2 diabetes mellitus (T2DM) and cardiovascular event risk [13, 14]. Recent studies also suggested that chemerin may play an important role in the pathogenetic mechanism of GDM. However, studies of the association between circulating chemerin levels and GDM yielded inconsistent findings. Therefore, we carried out a meta-analysis to provide a more comprehensive estimation of the association between circulating chemerin levels and GDM.

## Methods

### Search strategy

A systematic literature search was carried out in electronic databases including PubMed, EMBASE and Web of Science up to 13 December 2017. The search terms included: chemerin AND (“gestational diabetes mellitus” OR “gestational diabetes” OR “GDM”). In addition, the references from these relevant articles were manually searched for additional eligible studies.

### Inclusion and exclusion criteria

Original research evaluating the associations between circulating chemerin levels and pregnancy outcomes were considered eligible if they met the following criteria: (1) GDM as outcome and the control were healthy pregnant women with normal glucose tolerance (NGT); (2) all the subjects did not have a previous history of diabetes or present pregnant complications; or (3) studies were published in English or Chinese. Studies were excluded if they were letters to the editor, short report, conference abstracts, reviews, or studies on animals or cell lines.

### Data extraction

Two investigators (Zhongwei Zhou and Hongmei Chen) independently reviewed all identified studies and extracted the data using a predefined form, and confirmed by a third reviewer (Mingzhong Sun). Disagreement was resolved by discussion among all researchers. The following information was abstracted from each eligible study: the first author’s name, year of publication, study location, study design, trimester of chemerin measurement, average age and body mass index (BMI) of GDM patients, diagnostic criteria of GDM, sample size of the case and control group, mean and standard deviation (SD) of chemerin.

### Quality assessment

The quality of the study was evaluated using a modified criteria based on the Newcastle-Ottawa Quality Assessment Scale (NOS) for observational studies suggested by van Dijk et al. [15], The full score was 9 stars, and a study that met 7 or more stars would be considered as a high-quality study, less than 3 stars low-quality study, and other studies were defined as moderate quality.

### Statistical analysis

Standardized mean differences (SMD) and 95% confidence interval (CI) was calculated and estimated the differences in circulating chemerin levels between GDM patients and controls. A random-effect model which is more conservative than the fixed effect model was chosen for pooling of data [16]. Heterogeneity among studies was assessed using Cochran’s Q-test at *p* < 0.1, and quantified by the *I*^2^ index, and an *I*^2^ index of 25, 50 and 75% would indicate small, moderate and high heterogeneity, respectively [17]. Subgroup analysis was carried out to explore possible explanations for heterogeneity. Sensitivity analysis was performed to valuate the influence of a single on the pooled measures by omitting one study in each turn and recalculating the pooled SMD for the remainders. Publication bias was evaluated by inspection of funnel plots, and the Egger’s test. All analyses were performed using Stata14.0 (StataCorp LP, College Station, TX, USA) and *P* < 0.05 was considered to be statistically significant.

## Results

### Literature search

**Fig. 1:**
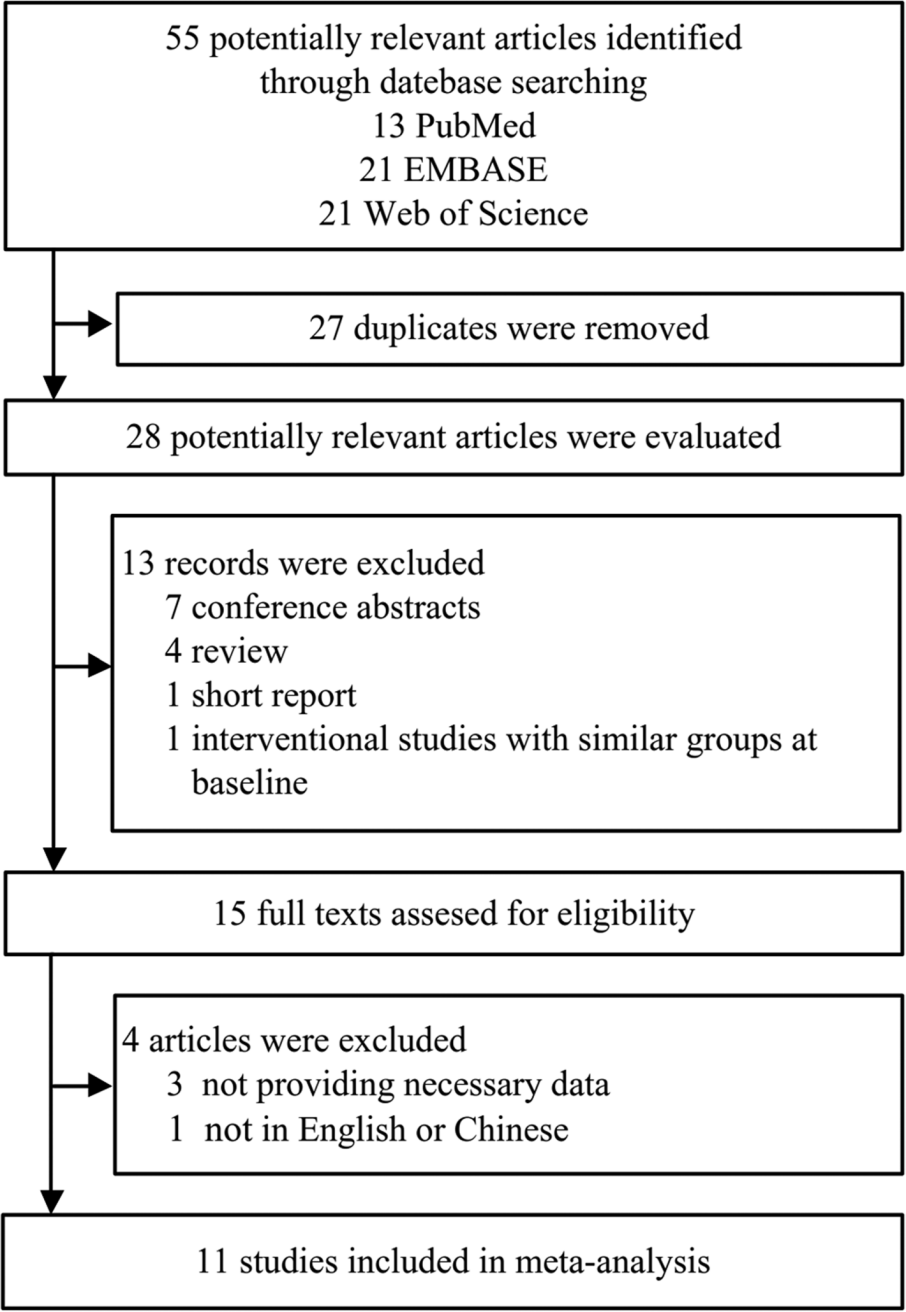
Flow chart of the study selection process

A flowchart of the included and excluded studies is showed in Fig. 1. A total of 55 records were identified after an initial search from the selected electronic databases. After removing duplicates and reading the titles and abstracts, 15 appropriate articles were identified for full text scrutiny. We further excluded 3 studies for lack of necessary data, and one study not in English or Chinese. Finally, 11 studies (13 results) met the criteria and were selected for the final analysis [18–28].

### Characteristics of the included studies

The 11 included studies were published from 2010 to 2017 covering 742 GDM patients and 840 normal pregnant women. The characteristics of the studies included in the present are presented in Table 1. Of the 11 included studies, all of them were cross-sectional, and ELISA methods were applied to measure circulating chemerin levels in all the studies. Five studies were carried out in China, two in Turkey, one in Pakistan, one in Australia, one in Germany and one in the Kingdom of Saudi Arabia (KSA). Seven studies measured circulating chemerin levels during the second trimester, five during the third trimester and one during the first trimester. GDM was diagnosed based on several different criteria, and among them, Fourth International Work-shopVconference on gestational diabetes (WC), The Australasian Diabetes in Pregnancy Society (ADIPS), The International Association of Diabetes and Pregnancy Study Groups (IADPSG), American Diabetes Association (ADA), Carpenter and Couston (C&C), Endocrine Society Clinical Practice Guideline (ESCPG), American College of Obstetricians and Gynecologists (ACOG) and World Health Organization (WHO) were used in different studies. The sample size of these studies ranged from 19 to 208 among GDM patients, and 20 to 300 among normal pregnant women. The range of mean chemerin levels was 1.65 to 308.6 ng/mL among GDM patients, and 1.57 to 227.5 ng/mL among normal pregnant women. The quality assessment results showed that two studies [20, 28] were assessed as high quality, and the other nine studies were all assessed as moderate quality, which was exhibited in Table 1.

**Table 1:** Main characteristics of the studies included in this meta-analysis

References	Study location	Study design	Case group		Control group		Chemerin measurement trimester	Average age of GDM patients (years)	Average BMI of GDM patients (kg/m^2^)	GDM criteria	Quality Score
			Sample size	Chemerin (ng/mL)	Sample size	Chemerin (ng/mL)					
Pfau, 2010 [18]	Germany	cross-sectional	40	230.3 ± 42.4	80	217.6 ± 72.3	Second	33.0	24.9	WC	3
Barker, 2012 [19]	Australia	cross-sectional	69	117.6 ± 29.1	62	124.2 ± 31.5	Third	35.2	28.2	ADIPS	6
Shao, 2015 [20]	China	cross-sectional	20	83.75 ± 15.88	20	77.80 ± 24.49	Third	30.0	28.73	IADPSG	7
Ademoglu, 2015 [21]	Turkey	cross-sectional	47	1.65 ± 0.17	32	1.57 ± 0.16	Second	30.2	31.3	ADA	4
Li, 2015 [22]	China	cross-sectional	48	194.0 ± 103.4	42	135.8 ± 89.3	Second	na	27.7	IADPSG	4
Görkem, 2016 [23]	Turkey	cross-sectional	76	3.64 ± 1.27	82	3.47 ± 0.61	Second	29.0	33.3	C&C	5
Pan, 2016 [24]	China	cross-sectional	85	9.12 ± 0.75	85	6.47 ± 0.59	Third	28.1	23.6	ADA	5
Fatim, 2017 [25]	Pakistan	cross-sectional	208	93.39 ± 45.43	300	14.35 ± 5.88	Second	27.3	24.8	IADPSG	4
Zhang, 2017 [26]	China	cross-sectional	60	11.2 ± 1.17	60	5.76 ± 0.03	Second	29.1	38.7	ESCPG & ACOG	3
Yang a, 2017 [27]	China	cross-sectional	19	146.6 ± 38.9	20	187.2 ± 46.8	First	26.8	22.7	IADPSG	6
Yang b, 2017 [27]	China	cross-sectional	19	308.6 ± 56.4	20	227.5 ± 46.5	Third	26.8	25.7	IADPSG	
Gashlan a, 2017 [28]	KSA	cross-sectional	25	7.21 ± 5.95	19	7.68 ± 5.93	Second	32.4	33.4	WHO	7
Gashlan b, 2017 [28]	KSA	cross-sectional	26	6.57 ± 4.84	18	5.56 ± 3.90	Third	33.4	34.1	WHO	

*GDM* gestational diabetes mellitus, *BMI* body mass index, *KSA* the Kingdom of Saudi Arabia, *WC* Fourth International Work-shopVconference on gestational diabetes, *ADIPS* The Australasian Diabetes in Pregnancy Society, *IADPSG* The International Association of Diabetes and Pregnancy Study Groups, *ADA*, American Diabetes Association, *C&C* Carpenter and Couston, *ESCPG* Endocrine Society Clinical Practice Guideline, *ACOG* American College of Obstetricians and Gynecologists, *WHO* World Health Organization, *na* not available

### Overall meta-analysis

**Fig. 2:**
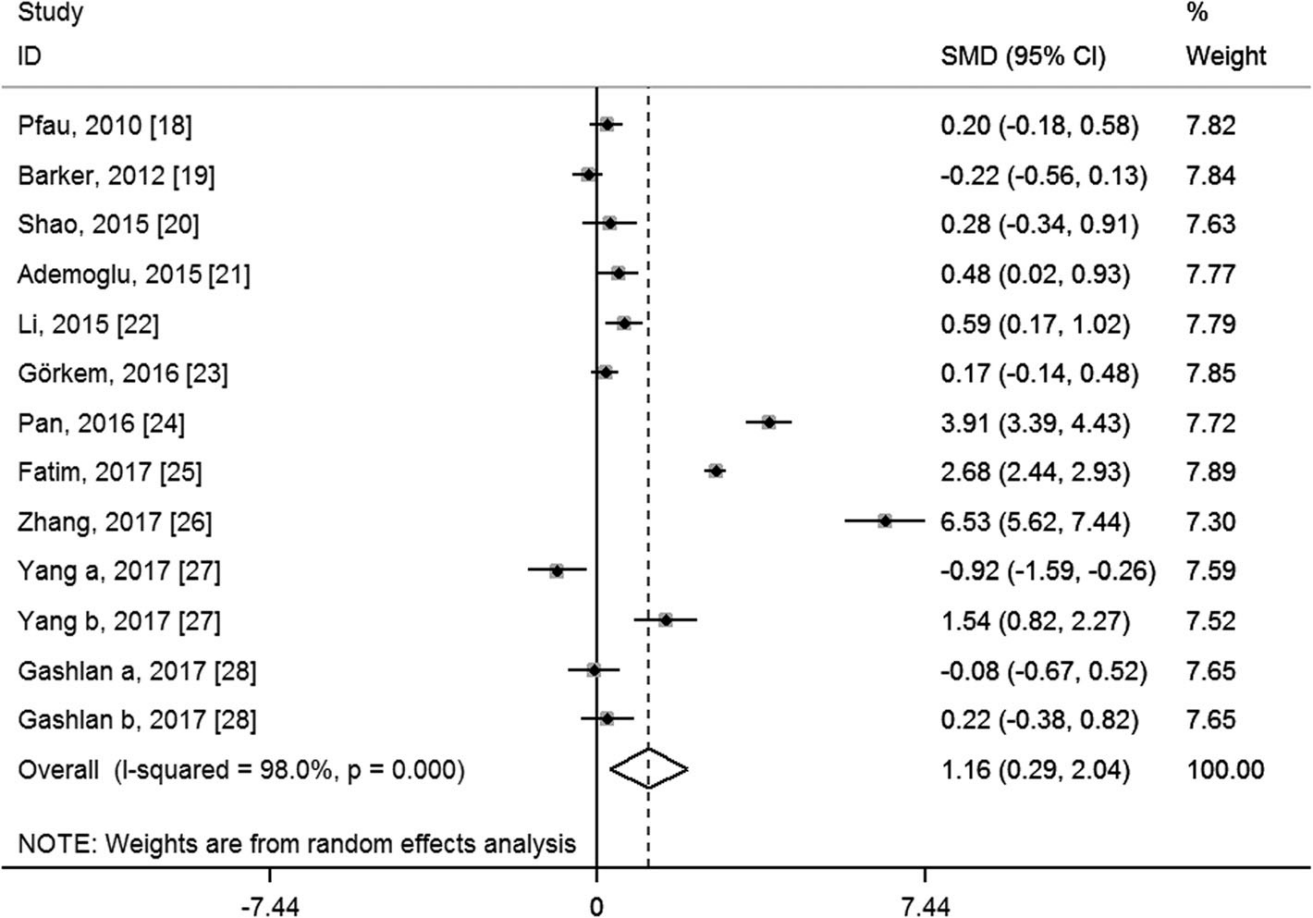
Overall meta-analysis of circulating chemerin levels in gestational diabetes mellitus patients compared with healthy pregnant women. SMD, standardized mean differences; CI, confidence interval

**Fig. 3:**
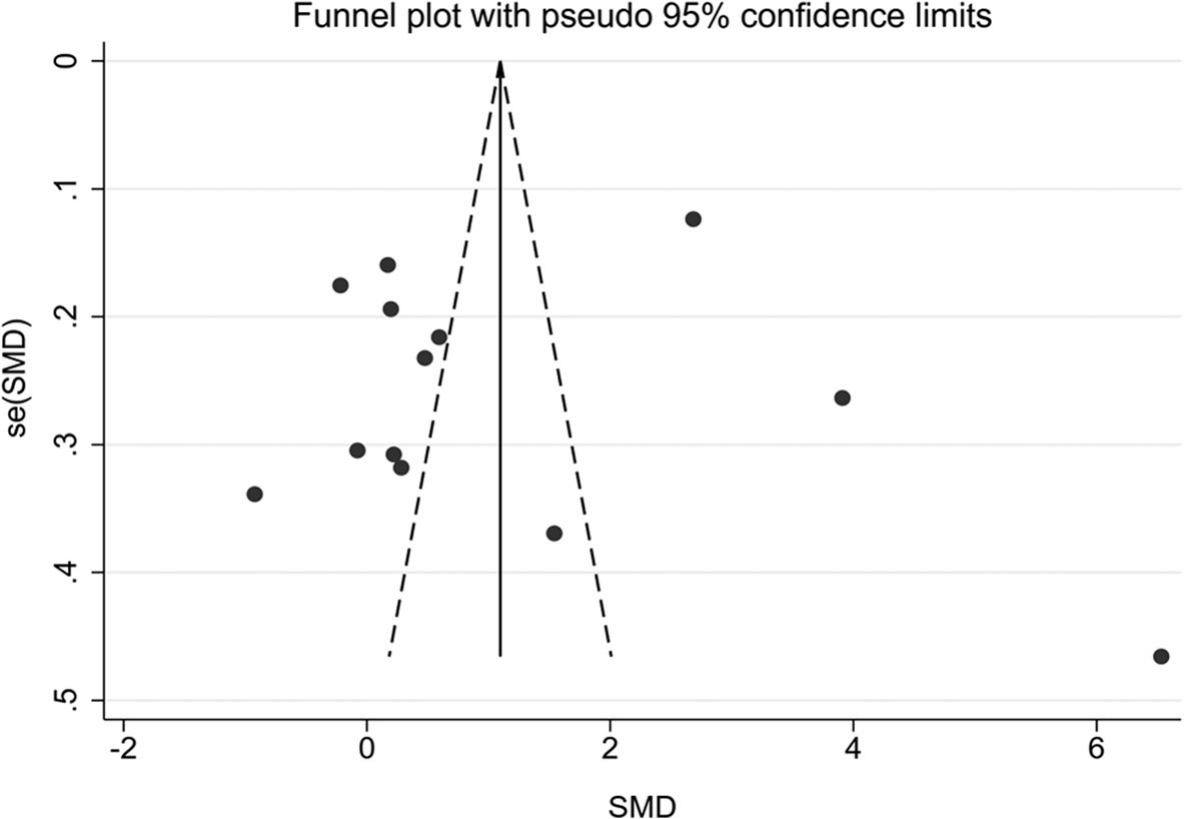
Funnel plot of included studies for potential publication bias between gestational diabetes mellitus patients and healthy pregnant women. SMD, standardized mean differences; se, standard error

We performed a random-effects meta-analysis on the extracted 11 studies. As indicated in Fig. 2, the overall levels of circulating chemerin in GDM patients were significantly increased (SMD: 1.16; 95% CI: 0.29, 2.04; *P* = 0.009) when compared with healthy pregnant women. Sensitivity analysis showed that no individual study had a significant effect on the circulating chemerin levels between GDM patients and healthy pregnant women. No significant publication bias was found in this meta-analysis by Egger’s test (*P* = 0.324), and the funnel plot with pseudo 95% confidence limits is shown in Fig. 3. However, significant heterogeneity among studies was found in this meta-analysis (*I*^2^ = 98.0%, *P* < 0.001).

### Subgroup analyses

**Fig. 4:**
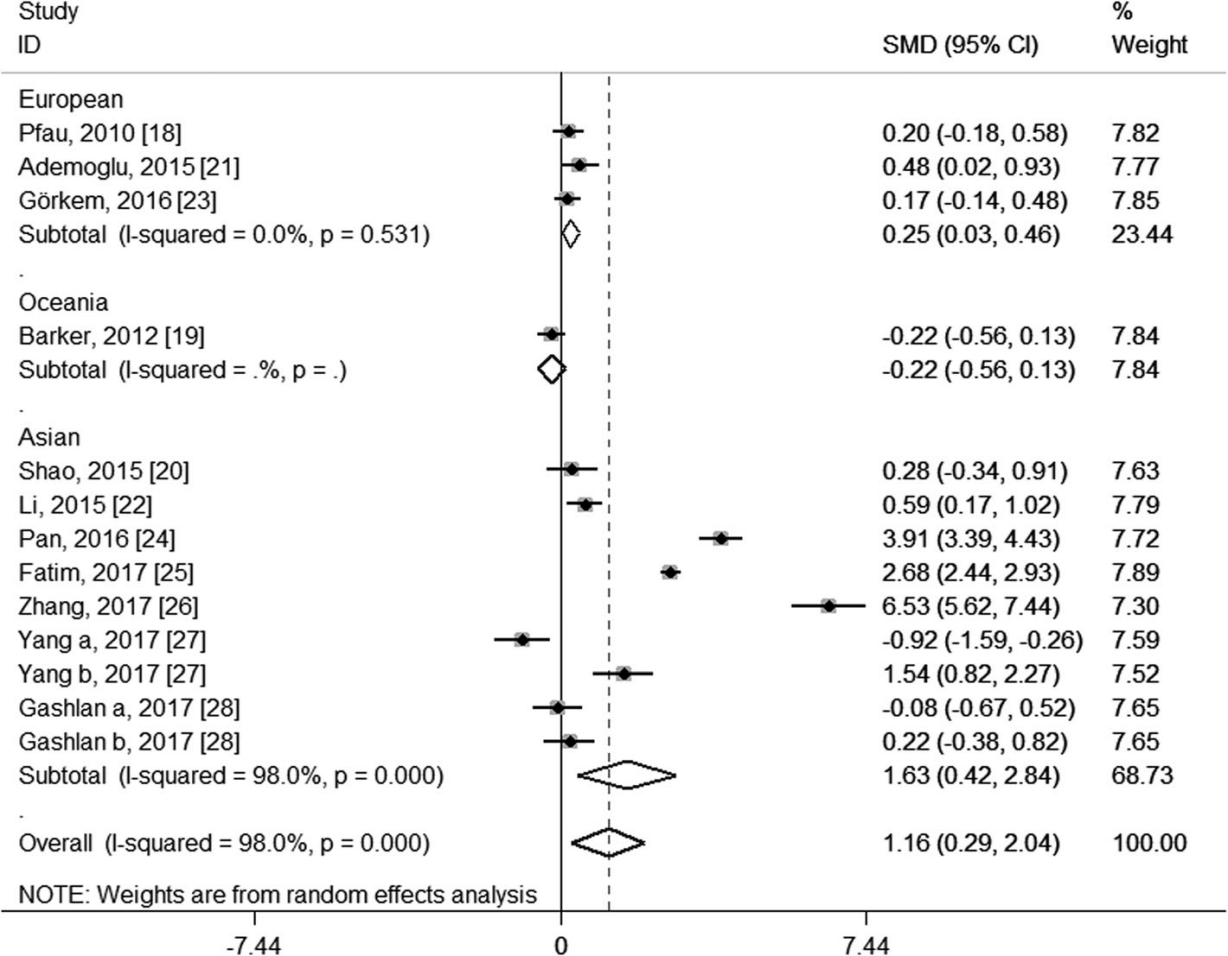
Subgroup analysis of circulating chemerin levels in gestational diabetes mellitus patients compared with healthy pregnant women when stratified by study location. SMD, standardized mean differences; CI, confidence interval

**Fig. 5:**
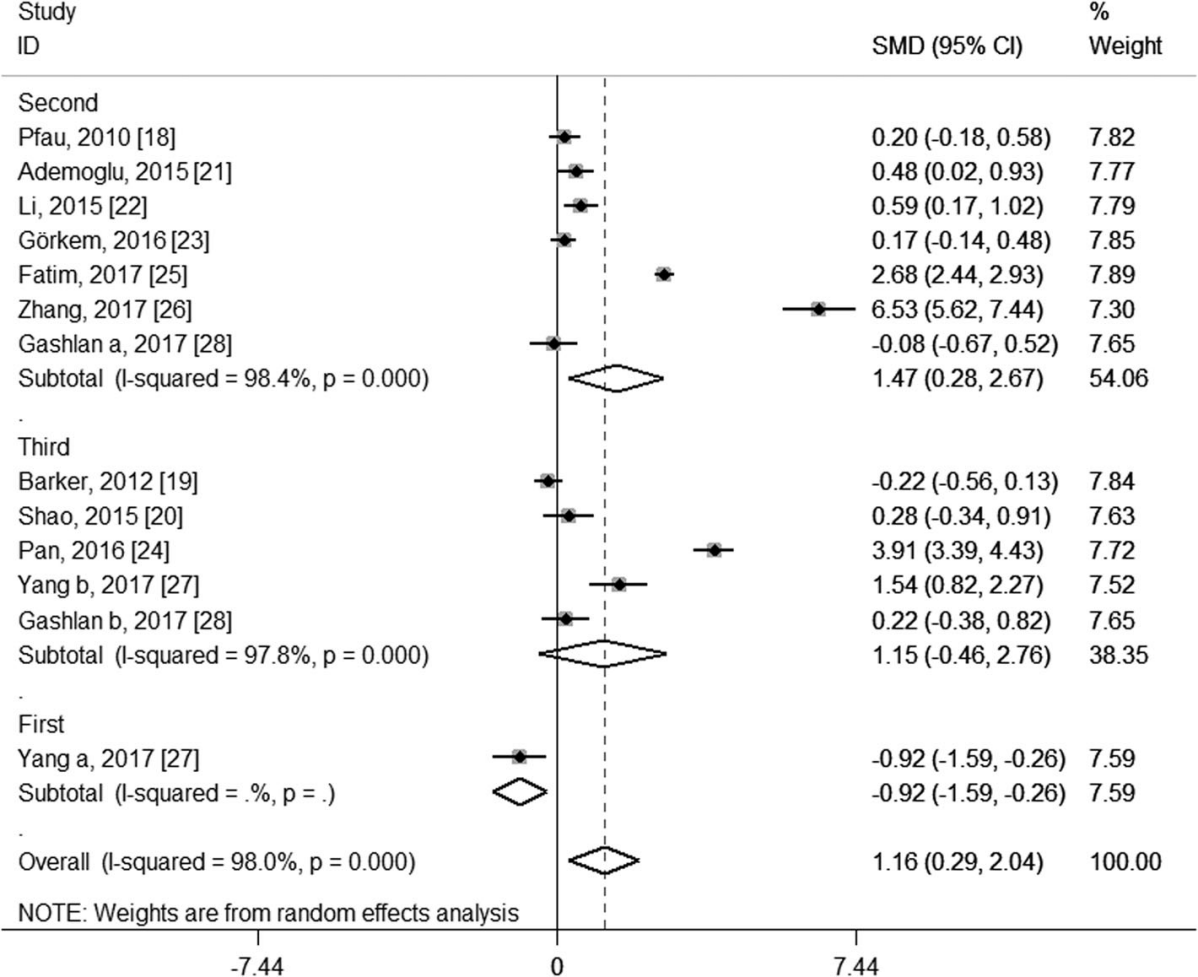
Subgroup analysis of circulating chemerin levels in gestational diabetes mellitus patients compared with healthy pregnant women when stratified by trimester of chemerin measurement. SMD, standardized mean differences; CI, confidence interval

**Fig. 6:**
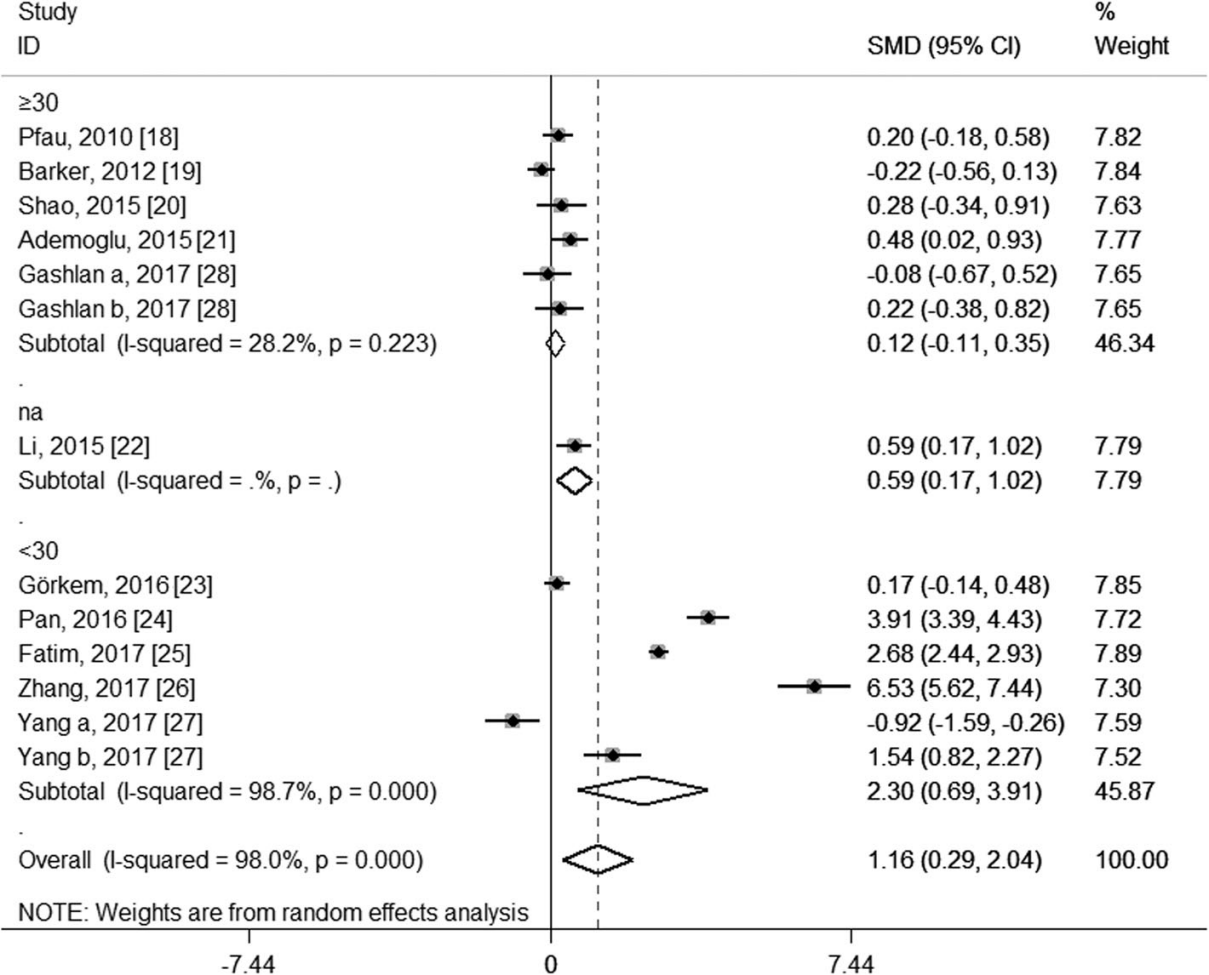
Subgroup analysis of circulating chemerin levels in gestational diabetes mellitus (GDM) patients compared with healthy pregnant women when stratified by the average age of GDM patients. SMD, standardized mean differences; CI, confidence interval; na, not available

**Fig. 7:**
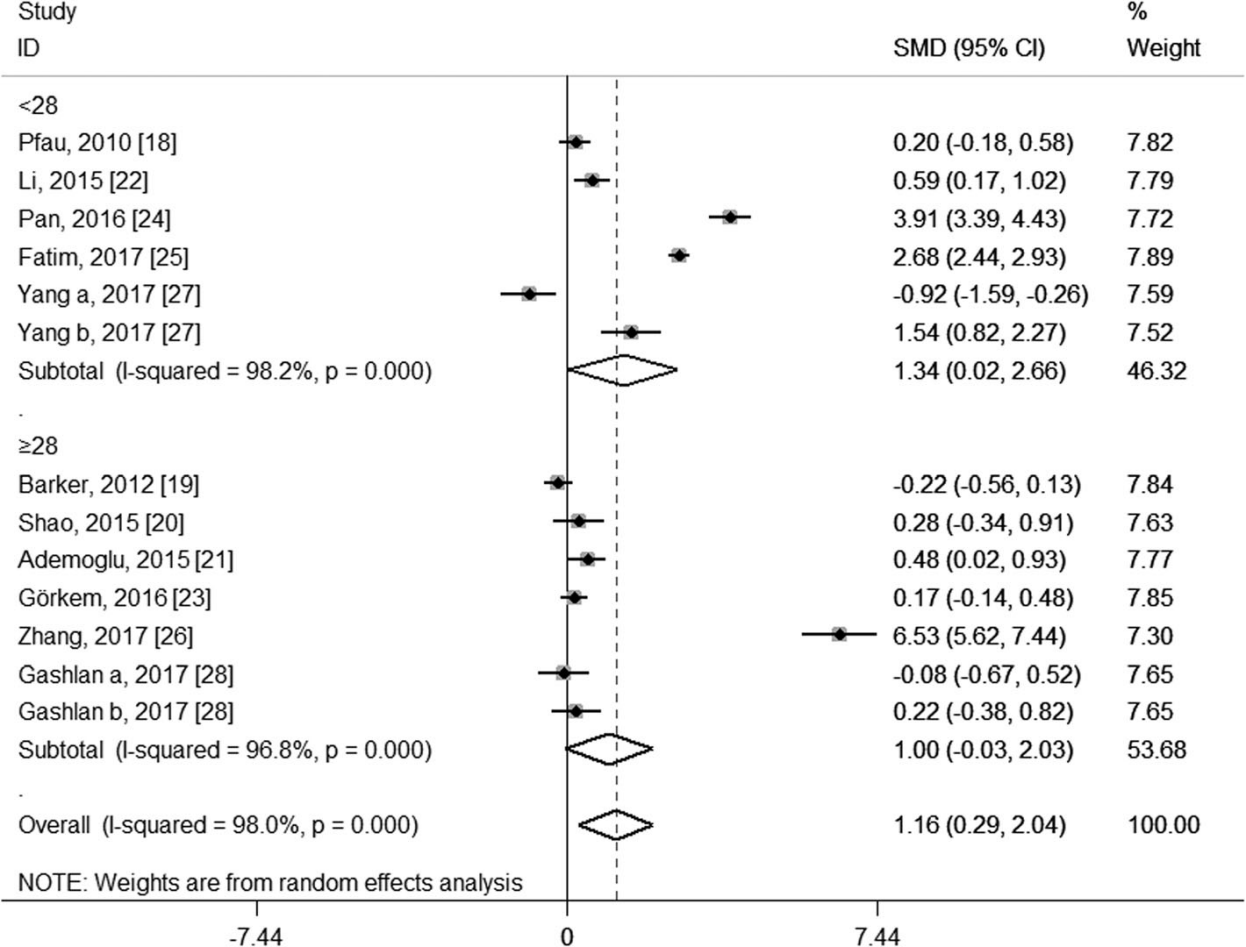
Subgroup analysis of circulating chemerin levels in gestational diabetes mellitus (GDM) patients compared with healthy pregnant women when stratified by mean body mass index of GDM patients. SMD, standardized mean differences; CI, confidence interval

**Fig. 8:**
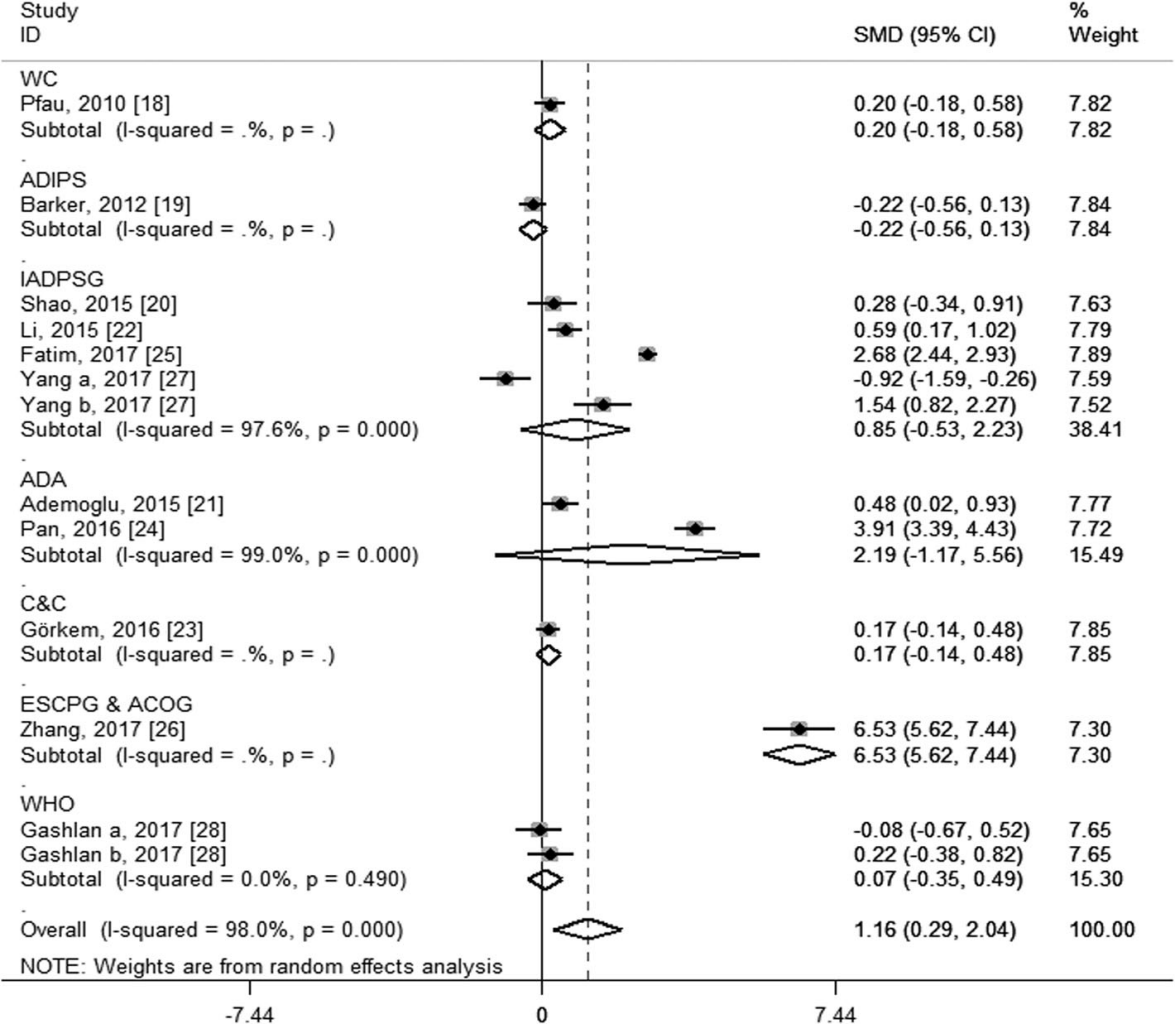
Subgroup analysis of circulating chemerin levels in gestational diabetes mellitus (GDM) patients compared with healthy pregnant women when stratified by the diagnostic criteria of GDM. WC, Fourth International Work-shopVconference on gestational diabetes; ADIPS, The Australasian Diabetes in Pregnancy Society; IADPSG, The International Association of Diabetes and Pregnancy Study Groups; ADA, American Diabetes Association; C&C: Carpenter and Couston; ESCPG, Endocrine Society Clinical Practice Guideline; ACOG, American College of Obstetricians and Gynecologists; WHO, World Health Organization; SMD, standardized mean differences; CI, confidence interval

To explore the potential source of the high levels of heterogeneity among studies, we performed subgroup analysis according to study location, trimester of chemerin measurement, the average age and BMI of the GDM patients and the diagnostic criteria of GDM. When stratifying by study location, these studies were classified as Asian group, European group and Oceanian group. As shown in Fig. 4, both the Asian group (SMD: 1.63; 95% CI: 0.42, 2.84) and the European group (SMD: 0.25; 95% CI: 0.03, 0.46) showed higher circulating chemerin levels among women with GDM, especially in the Asia; and although no heterogeneity was observed in the European group (*I*^2^ = 0.0%, *P* = 0.531), considerable heterogeneity were still found in the Asian group (*I*^2^ = 98.0%, *P* < 0.001). When stratifying by trimester of chemerin measurement, the second-trimester patients showed significantly higher levels of circulating chemerin compared with healthy pregnant women (SMD: 1.47; 95% CI: 0.28, 2.67), while the difference did not reach statistical significance during the third trimester (SMD: 1.15; 95% CI: − 0.46, 2.76), and GDM patients in the first trimester showed even significantly decreased chemerin levels than those in healthy pregnant women (SMD: − 0.92; 95% CI: − 1.59, − 0.26); however, significant heterogeneity among studies was still observed in the second group (*I*^2^ = 98.4%, *P* < 0.001) and the third trimester group (*I*^2^ = 97.8%, *P* < 0.001) (Fig. 5). In the subgroup analysis of the average age, GDM patients with mean age < 30 years had significantly increased circulating chemerin levels when compared with controls (SMD: 2.30; 95% CI: 0.69, 3.91); however, the difference was not significant between patients with mean age ≥ 30 years and controls (SMD: 0.12; 95% CI: − 0.11, 0.35); significantly decreased heterogeneity was observed in the group of age ≥ 30 years (*I*^2^ = 28.2%, *P* = 0.223), but not in the group of age < 30 years (*I*^2^ = 98.7%, *P* < 0.001) (Fig. 6). In stratified analyses based on mean BMI of GDM patients, the difference of the chemerin levels between the GDM patients and controls was more significant in the group of mean BMI < 28 (SMD: 1.34; 95% CI: 0.02, 2.66) than that of mean BMI ≥28 (SMD: 1.00; 95% CI: − 0.03, 2.03), but dramatic heterogeneity were still observed in both groups (*I*^2^ = 98.2 and 96.8% respectively; *P* < 0.001) (Fig. 7). When stratifying by the diagnostic criteria of GDM, patients defined by ESCPG & ACOG criteria had significantly higher chemerin levels compared with control (SMD: 6.53; 95% CI: 5.62, 7.44), but those defined by other criteria had not; although no heterogeneity was observed in the WHO subgroup (*I*^2^ = 0.0%, *P* = 0.490), significant heterogeneity were still found in the IADPSG subgroup (*I*^2^ = 97.6%, *P* < 0.001) and ADA subgroup (*I*^2^ = 99.0%, *P* < 0.001) (Fig. 8).

## Discussion

This meta-analysis demonstrated that the circulating chemerin levels was significantly increased in women with GDM than healthy pregnant women. Sensitivity analysis showed that the pooled results were not unduly influenced by a particular study. In addition, we did not find significant publication bias in this meta-analysis. To the best of our knowledge, this is the first meta-analysis on this subject, which suggests chemerin may play an important role in the pathogenetic mechanism of GDM.

In the meta-analysis, subgroup analysis was used to analyze the potential factors contributing to heterogeneity and obtain further information from different sub-populations. When stratifying by study location, average age of the GDM patients and the diagnostic criteria of GDM, we found heterogeneity disappeared or markedly decreased in the subgroups of European populations, age ≥ 30 years and WHO diagnostic criteria, which suggests study location, age of GDM patients and the diagnostic criteria of GDM may be associated with heterogeneity at some level. In addition, we found the significant difference of circulating chemerin levels between GDM patients and healthy pregnant women was especially available in the subgroups of the second trimester but not the third trimester. In line with the pooled results, one studies [28] included in the present meta-analysis demonstrated that both GDM patients and healthy pregnant women had higher chemerin levels in the second trimester than those of corresponding groups in the third trimester. Recent research indicated that adipokines such as chemerin are released from human serum albumin, apart from human adipose tissue [29]; and serum albumin levels are usually decreased in late pregnancy because of increasing nutrition needs of fetus, which may partly account for the lower levels of circulating chemerin for GDM patients during the third trimester measurement. But this phenomenon that the significant difference of chemerin levels was observed in the second-trimester patients may be the result of more studies included in the subgroup. Therefore, further large-scale investigations are needed to confirm this result and expound its significance. We also observed the significant difference of the comparison of GDM vs controls in the subgroups of mean age < 30 years rather than ≥30 years, suggesting that there may be a negative correlation between age and circulating chemerin levels in GDM patients. However, there is little research involved in the relationship of age with chemerin in women with GDM. Two recent studies reported that there were weakly negative correlations between age and serum chemerin levels in T2DM patients, but which was not significant [30, 31]. Thus more research is also needed to confirm their link in GDM patients. In this meta-analysis, we did not find higher levels of circulating chemerin in higher BMI group, although previous research has indicated that elevated chemerin expression was associated with obesity and obesity-related disease. The possible explanation is that BMI is not an ideal index for reflecting obesity, as it balances both height and weight that reflect the total body fat. However, the indexes reflecting central obesity such as waist circumference and waist-hip ratio cannot be acquired in studies included in this meta-analysis.

In recent years, an increasing number of studies have found that a variety of adipokines may be involved in the pathophysiology of GDM. A review in 2014 by Fasshauer et al. [32] provided an overview of various adipokines in GDM, and suggested adiponectin, leptin, tumour necrosis factor α (TNFα) and adipocyte fatty acid-binding protein (AFABP) are more likely than chemerin and other adipokines to play roles in the pathogenic mechanism of GDM based on previous reports. In this paper, we collected 11 studies which were systematically and quantitatively analyzed and provided a more comprehensive estimation that chemerin may play a powerful role in the pathophysiology of GDM. The mechanism that chemerin involves in endocrine and metabolic regulation is highly intricate because of its several isoforms, differential expression in different organs and the diverse activation mechanism [8, 33 32]. As the pathophysiologic mechanism of GDM is similar to T2DM in which insulin resistance and chronic inflammation are the most important pathophysiological basis, and elevated chemerin expression has been shown to contribute to the development of insulin resistance and low-grade chronic inflammation [8–10], it is possible that chemerin involved in the pathophysiologic mechanism of GDM is by increasing insulin resistance and promoting subclinical inflammation. Several studies included this meta-analysis also indicated that GDM patients had significantly higher levels of homoeostasis model assessment of insulin resistance (HOMA-IR) and inflammatory parameters such as C-reactive protein when compared with healthy pregnant women [22–26], and there were significant positive correlations between serum chemerin levels and insulin resistance, and inflammatory parameters [18, 22, 24].

There are several limitations in this met-analysis. First, remarkable heterogeneity among studies limits the reliability of the results. Although subgroup analyses were used to explore the potential sources, the high levels of heterogeneity cannot be sufficiently and reasonably explained. Moreover, as data on some potential confounder such as exercise, diet adjustment and the levels of inflammation and insulin resistance are limited in the eligible studies included in the meta-analysis, we cannot analyze whether these factors had moderating effects on the pooled results. Therefore, the results of this meta-analysis should be cautiously interpreted. Second, all of the studies included in this meta-analysis were cross-sectional, thus a causal relationship between circulating chemerin levels and GDM cannot be established. Third, the publication bias may not be avoided absolutely although Egger’s test was applied in this analysis, as only published studies in English and Chinese in the selected databases were included. Finally, the quality assessment of studies included in this meta-analysis was based on the modified NOS due to the lack of appropriate assessment tool for cross-sectional studies, which might result in arbitrary results [33, 34].

## Conclusions

In conclusion, this meta-analysis demonstrated that the elevated levels of circulating chemerin were associated with GDM, which suggests it might play an important role in the pathogenetic mechanism of GDM. However, the results should be cautiously interpreted owing to substantial heterogeneity among studies, and further prospective cohort studies are required to confirm these findings.
